# Adventitious Bursitis Overlying an Osteochondroma of the Humerus Facing the Thoracic Wall

**DOI:** 10.1155/2013/939372

**Published:** 2013-11-28

**Authors:** Zeynep Maras Ozdemır, Mustafa Karakaplan, Aysegul Sagir Kahraman, Nese Karadag

**Affiliations:** ^1^Department of Radiology, Inonu University School of Medicine, Turgut Ozal Medical Center, 44280 Malatya, Turkey; ^2^Department of Orthopedics and Traumatology, Inonu University School of Medicine, 44280 Malatya, Turkey; ^3^Department of Pathology, Inonu University School of Medicine, 44280 Malatya, Turkey

## Abstract

One of the complications of osteochondromas is the development of a bursa over the cartilaginous cap. We report a 15-year-old boy with a rapidly expanded adventitious bursitis overlying an osteochondroma of the humerus facing the thoracic wall, a location not previously reported for such bursa formation. Magnetic resonance imaging readily showed adventitious bursitis overlying the osteochondroma, thereby dispelling concerns for malignant transformation.

## 1. Introduction

One of the complications of osteochondromas is the development of a bursa over the cartilaginous cap. Differential diagnosis of a rapid enlargement of the bursa overlying an osteochondroma and malignant transformation of the cartilaginous cap of osteochondroma is important because a rapid enlargement of the bursa may be interpreted as a malignant transformation [1]. We describe a large adventitious bursa formation related with an osteochondroma of the humerus. Although adventitious bursa formation on osteochondromas was reported in the upper extremities, a bursa formation in the humerus was not reported before.

## 2. Case Report 

A right-hand-dominant 15-year-old boy was followed since five years of age for a slow-growing solitary osteochondroma in the right humerus. He presented with a rapidly increased swelling at the site of the lesion over the course of a month. There was no pain or neurovascular symptoms. He had a history of recent trauma to the region of concern while playing with his older brother. Physical examination revealed a nontender, painless, fluctuating soft tissue mass at the anteromedial side of the proximal right arm. The right shoulder of the patient had no limitations during the active range of motions. Laboratory findings were normal.

**Figure 1:**
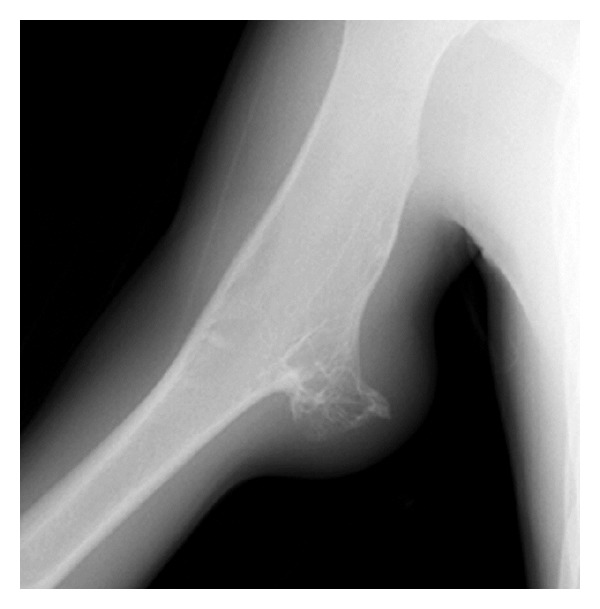
Oblique radiograph of the right arm shows soft tissue swelling overlying a broad-based osteochondroma arising from the humerus and directed towards the chest wall.

**Figure 2:**
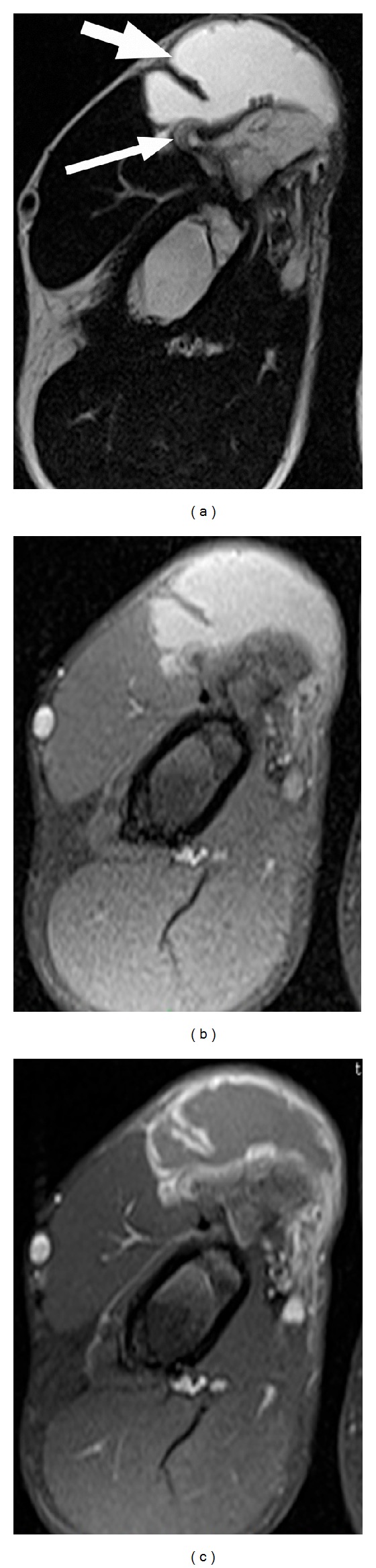
Axial T2-weighted (a), T1-weighted fat-suppressed precontrast (b), and postcontrast (c) MR images show an adventitious bursa *(thick arrow)* overlying the osteochondroma of the humerus. Note the thin cartilage cap *(thin arrow)*.

Anteroposterior oblique plain radiographs demonstrated a broad-based osteochondroma originating from the proximal diaphysis of the right humerus and an increased density of the overlying soft tissue. The expansion of the osteochondroma was towards the chest wall (Figure 1). A 3.5 × 3 × 2 cm, broad-based osteochondroma of the right humerus associated with an overlying fluid-containing sac showing peripheral enhancement on the postcontrast images suggestive of an adventitious bursitis was visualized on MRI (Figure 2). Cartilage cap of the osteochondroma was 3 mm in thickness and could be differentiated from the overlying cystic sac. Following nonsteroidal anti-inflammatory drug therapy, soft tissue swelling at the site of osteochondroma subsided.

**Figure 3:**
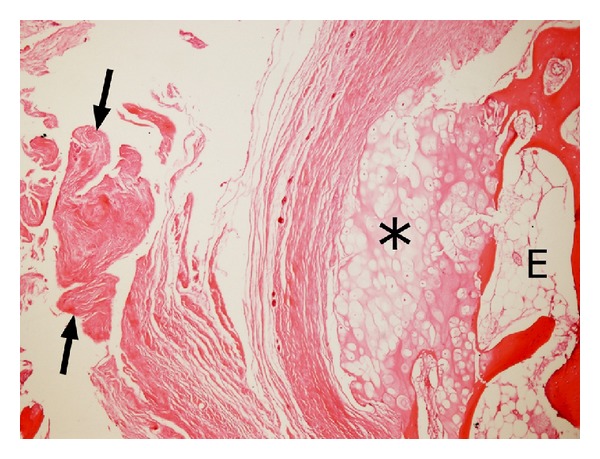
On histology, the bursal wall *(arrows) *overlying the cartilage cap *(asterisk)* of the osteochondroma (E) was composed of a dense fibrous tissue; there was no malignant transformation of the osteochondroma (HE, ×4).

At surgery, osteochondroma was covered by a bursal pouch that did not contain significant fluid. Resection of the tumor from the anteromedial surface of the proximal humerus was performed after the excision of hypertrophic bursal wall. The thickness of the cartilaginous cap of the lesion was 2-3 mm and the cap had a smooth surface. The bursal wall was 7-8 mm in thickness and somewhat irregular. There was not any diagnostic feature of malignant transformation of the osteochondroma on histological study; the bursal wall was composed of a dense fibrous tissue (Figure 3).

## 3. Discussion

A reactive bursa formation is relatively common complication of osteochondroma and arises from the friction between the osteochondroma and overlying soft tissue [1]. Although the scapula is a relatively rare location for solitary osteochondroma, reactive bursa formation overlying an osteochondroma is most commonly seen with lesions at the ventral aspect of the scapula [1]. Such bursa formation is also reported in association with osteochondromas of the femur, rib, ischium, and pubis [2–4]. To the best of our knowledge, however, no other case of bursa formation overlying an osteochondroma of the upper extremities has been published in the literature.

In the present case, the bursa was located at the anteromedial side of proximal right arm. This location on the dominant side with close proximity to the anterior thoracic wall is prone to repeated mechanical friction trauma during daily activities and might have contributed to the development of an adventitious bursa.

A painful and rapidly enlarging bursa clinically mimics a malignant transformation, particularly at an adult age when skeletal maturation has been completed. It is very important to differentiate these two complications, and this can be easily done by using the appropriate imaging modality, which is usually ultrasound or MRI [1–4].

Malignant transformation occurs within the cartilage cap of osteochondroma. Therefore, prediction of malignancy is largely based on the measurements of cartilage cap thickness, which can be assessed with ultrasound, CT, and MRI [5–7]. In two previous studies, cartilage cap thickness ranged between 0.1–3.0 cm (means, 0.6 and 0.8 cm) and 1.5–12 cm (means, 5.5 and 6.0 cm) for benign and malign lesions, respectively [5, 6]. Therefore, a cartilage cap thickness greater than 1.5 cm is strongly suggestive of malignancy in adult patients who completed their skeletal maturation [1].

A recent retrospective study reevaluated the relation between pathological findings and standardized measurements of cartilage cap thickness using CT and MRI, in an attempt to provide improved radiological differentiation between benign osteochondroma and chondrosarcoma. This study reported a cartilage cap thickness larger than 2 cm as a powerful indicator of secondary chondrosarcoma [7]. However, accurate measurements of cartilage cap thickness may be challenging in a case with bursa formation in association with osteochondroma since the bursal fluid and the cartilage cap with high fluid content have similar CT densities and similar MRI intensities on most MRI sequences [3, 7]. Cartilage cap measurements may be reliably performed at MRI sequences sensitive to fluid; in addition, proton density and gradient MRI sequences far better delineate the cartilage cap and the fluid collection than does CT. Nevertheless, it may be difficult to differentiate these two conditions with MRI sequences when the cartilage cap is thick [7]. Postcontrast MR images do not seem to improve differential diagnosis much since both the bursal wall and the cartilaginous cap would show similar peripheral and septal contrast enhancement [1, 7]. In addition, the difference between the bound water in the cartilage cap and free water in the bursal fluid can be demonstrated easily by the cartilage sensitive pulse sequences such as fat-suppressed three-dimensional spoiled gradient-recalled (SPGR) sequence, thereby cartilage cap thickness can be measured reliably on MRI [7, 8].

In summary, we described a patient with a rapidly enlarged adventitious bursitis overlying an osteochondroma of the humerus facing the thoracic wall, a location not previously reported for such bursa formation and where mechanic stresses related to extremity motion might have played a role. MRI is essential in differentiating adventitious bursa formation from malignant transformation in osteochondromas.
